# A Scoping Review of Student Pharmacist Participation on Interprofessional Rounds

**DOI:** 10.7759/cureus.58737

**Published:** 2024-04-22

**Authors:** Alice N Hemenway, Heidi R Olson

**Affiliations:** 1 Department of Pharmacy Practice, University of Illinois Chicago, Rockford, USA

**Keywords:** pharmacy education, scoping review, clinical rounding, interprofessional rounding, experiential education

## Abstract

Interprofessional rounding is a common, yet unrequired, part of the pharmacy experiential curriculum. Little is known about the optimal interprofessional rounding structure for student pharmacists. A scoping review was performed to assess the amount and type of information available regarding student pharmacist participation on interprofessional rounding teams. A comprehensive review of five databases was completed through May 12, 2023. A total of 20 studies met the inclusion criteria. All of the assessments performed were quasi-experimental, and the majority were non-comparative studies that described the type and amount of student interventions. A review of outcomes found that all of the studies could be grouped into two overarching categories: those that assessed the benefits of interprofessional rounding to student pharmacist competencies or satisfaction and those that assessed the benefits of student pharmacists to patient care. The benefits of interprofessional rounding on student pharmacist learning and satisfaction were assessed by qualitative analysis, surveys, and student ability assessments. The benefit of student pharmacist participation in interprofessional rounds to patient care was assessed solely by a review of clinical intervention type and quantity. Thirteen of the studies described the frequency of student pharmacist participation in rounding. Of these studies, eight described daily rounding, and five described non-daily rounding. There are few studies that describe student pharmacist participation on interprofessional rounds and assess the benefits of that participation to either the patient or the student. There is a need for more high-quality studies to determine whether there is an optimal interprofessional rounding schedule.

## Introduction and background

Interprofessional work is a cornerstone of healthcare, specifically of pharmacy practice. It is a required component of a pharmacy curriculum and is part of the Accreditation Council of Pharmacy Education (ACPE) standards [1]. Interprofessional and team-based work is also part of the Interprofessional Education Collaborative (IPEC) core competencies, as well as the Curriculum Outcomes and Entrustable Professional Activities (COEPA) recommendations [2-3]. Pharmacy is not the only profession to include interprofessional education as a standard. It is also part of the 2021 Accreditation Commission on Colleges of Medicine (ACCM) medical school standards [4].

A large part of interprofessional work is done within the experiential portion of the pharmacy curriculum, specifically on introductory pharmacy practice and advanced pharmacy practice experiences (IPPE, APPE). Since clinical rounding is a requirement of the ACCM standards and is a common occurrence at teaching hospitals, interprofessional rounding is often a good option for pharmacist preceptors seeking to provide an interprofessional team experience for students on inpatient APPE [4]. Although the benefits of interprofessional collaboration are known through anecdotal experiences, there is a limited amount of quality data showing benefits for interprofessional rounding teams on patient care [5]. Despite this lack of data, it remains the standard in many hospital systems, especially those associated with medical colleges or universities. However, interprofessional rounding teams are not universal and are often not a part of small or rural hospital practice [5-6]. While there is considerable literature for medical students on clinical and interprofessional rounds, there is no corresponding review for pharmacy students [7].

The ACPE standards, as well as the IPEC and COEPA guidelines, do not specify interprofessional rounding as a requirement [1-3]. Interprofessional rounding is a method that incorporates various healthcare professions to provide patient care in a simultaneous fashion. It is not the only way for students to gain interprofessional and teamwork experience, with data supporting brief interprofessional shadowing experiences, simulation activities, and other structured interprofessional activities [6,8]. The lack of standardization often leads to uncertainty for potential preceptors, with questions such as "How do I provide the best experience?" and "Should I design my rotation similar to how I was trained or try something different?". Without data, often old routines are continued. In order to provide guidance to preceptors seeking to try innovative interprofessional rounding techniques, and to help provide data to the dogma, this scoping review was performed to collect and analyze all available data on outcomes associated with student pharmacists on interprofessional rounds. 

## Review

Methods

The Preferred Reporting Items for Systematic Reviews and Meta-Analyses Extension for Scoping Reviews (PRISMA-SCr) guidelines were used for the design and reporting of the review. Since a limited amount of published data was anticipated, a scoping methodology was utilized to incorporate literature from a wide variety of sources, including grey literature. A systematic search of six databases, MEDLINE (Ovid), EMBASE (Ovid), Web of Science (Clarivate), ERIC (Ebsco), and Academic Search Complete (Ebsco), was performed between May 9 and 12, 2023, using a combination of interprofessional rounding terms. These terms were selected to answer the research question: "What evidence is available that supports pharmacy student participation on interprofessional rounds?". The MEDLINE search strategy is provided in the Appendices. The other databases were searched using the same combination of terms, which included terms related to clinical rounds and pharmacy students. These databases were chosen to provide a thorough review of medical and educational databases and have been used in other pharmacy education-based scoping reviews [9]. In addition, the reference lists for each of the final included publications were reviewed.

Articles that described results from studies assessing student pharmacists on interprofessional rounding teams were included, for all dates prior to the search dates. In anticipation of limited published data, an expansive definition of interprofessional rounding, defined as the pharmacy student and one other profession, was used. Anticipating limited randomized controlled trials, there were no exclusion parameters based on the type of study. Non-English language manuscripts, manuscripts that did not include an evaluative component, studies that assessed simulation-based activities, studies that did not include in-person, interprofessional clinical rounding activities, and studies that did not involve student pharmacists were excluded.

Title and abstract review was first performed for every database by both authors to remove sources that did not meet the inclusion criteria or were duplicates. After the initial title and abstract review, the full text of eligible articles was screened by both authors according to the same exclusion criteria. Any discordant results were discussed between both of the authors, with a decision made by consensus after a second review of the study. Data extraction was performed using a coding guide that included information on study design, setting, participants, rounding details, and outcomes. Discrepancies in data interpretation were discussed between both authors until a consensus was obtained. Study-based synthesis was limited due to the few number of studies found.

Results

**Figure 1:**
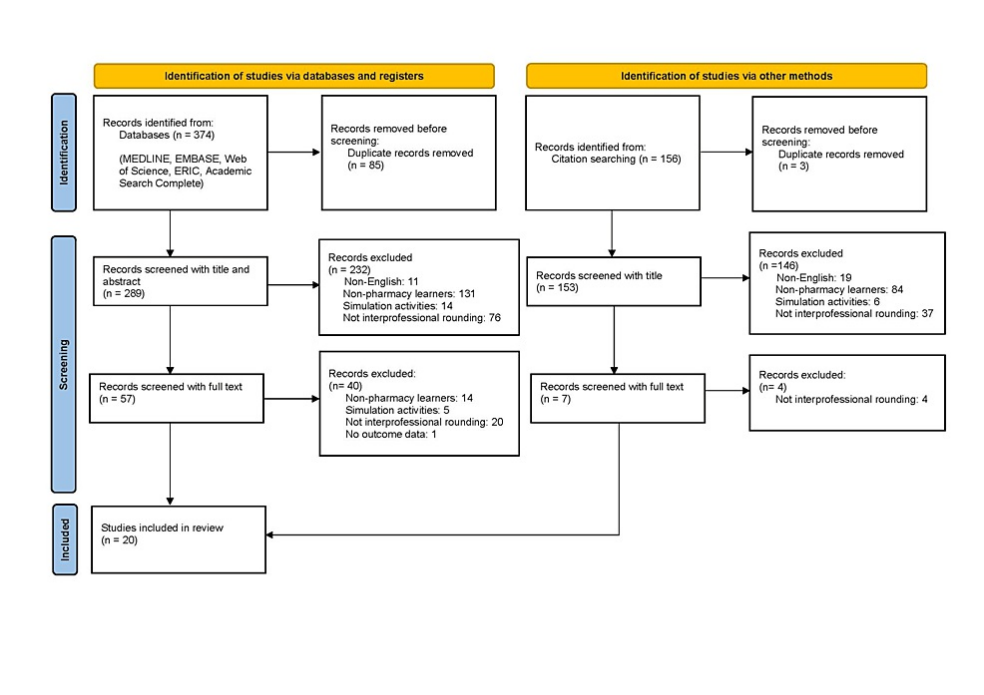
Scoping review flow diagram

After a review of all databases and reference lists for the included studies, 530 articles were found. After the removal of duplicates, 442 articles remained. After the two rounds of review, 20 articles were included in the final results (4.5% of the total minus duplicates). The majority of articles were excluded because they did not include student pharmacist learners. The PRISMA diagram is available in Figure 1. A summary of the included articles is available in Table 1 [10-29].

**Table 1:** Studies that describe student pharmacist participation on interprofessional rounds a: benefit to student learning or satisfaction; b: benefit to patients or practice site

Study	Rounding details	Setting and population	Evaluation methods (a, b)	Outcomes and conclusions
Al-Hajje et al. (2012) [10]	Inpatient, unit-based internal medicine team consisting of physician, pharmacist, and student pharmacist. Frequency and duration of rounds not noted	Non-US, single-site, university-affiliated, tertiary-care hospital	Prospective, non-comparative study evaluating the type and quantity of student interventions (b)	Intervention acceptance rate of 96%; the most common type of intervention was drug interaction; the most common drug class was cardiovascular
Chisholm et al. (1997) [11]	Inpatient, general medicine or family medicine teams consisting of physician, resident physician, and student pharmacist. Daily rounds, with 25% of activities in outpatient setting	Single-site, university-affiliated, tertiary-care hospital in Georgia	Prospective, non-comparative study evaluating the type and quantity of student interventions (b)	Average intervention acceptance rate of 86%; 70% of accepted recommendations were considered significant to patient care
Dennehy et al. (1998) [12]	Inpatient rounding consisting of physician, resident pharmacist, and student pharmacist. No explicit mention of frequency or duration of rounds	Multi-site, university-affiliated hospitals in California	Prospective, non-comparative study evaluating the type and quantity and outcome of student interventions (b)	Students performed an average of 1.8-6.2 interventions per week, and the average increased throughout the rotation
Eudaley et al (2022) [13]	Inpatient family medicine team consisting of physician, pharmacist, resident physicians, resident pharmacist, and student pharmacist. Frequency or duration of rounds not noted	Community, teaching, tertiary-care hospital in Tennessee	Retrospective pre-/post-survey to assess changes in perceptions of autonomy for five entrustable professional activities (a)	Student perception of autonomy increased for the five entrustable professional activities assessed
Foral et al. (2016) [14]	Inpatient antimicrobial stewardship team consisting of physician, pharmacist physician fellow, resident physician, resident pharmacist, student physician, and student pharmacist. Daily rounds	Multi-site (two), university-affiliated hospitals in Nebraska	Non-comparative study evaluating the type and acceptance rates of antimicrobial stewardship interventions (b)	The acceptance rate for interventions was 90% and was highest (96%) for the route of administration changes; the most common intervention type was to streamline therapy
Helmer et al. (2020) [15]	Outpatient team consisting of physician, pharmacist, resident physician, and student pharmacist. Frequency or duration of rounds not noted	Multi-site, ambulatory care clinics in Alabama	Pre-/post-student self-evaluation and pre-/post-survey assessing student confidence in interprofessional communication and comfort level with rounding (a)	Self-evaluation scores increased in all areas except professional language; comfort level with rounding increased
Hemenway and Meyer-Junco (2022) [16]	Inpatient internal medicine teams consisting of physician, resident physicians, student physicians, and student pharmacist. Three-day-a-week rounds, for approximately three hours per day	Single-site, community, teaching hospital	Retrospective post-survey assessing student self-reported confidence and knowledge from the three-day-a-week rounding schedule (a)	Responses or "positive impact" or "extreme positive impact" ranged from 67% to 100%
Jones et al. (2011) [17]	Inpatient internal medicine or family medicine teams consisting of physician, pharmacist, resident physician, and student pharmacist. Frequency or duration of rounds not noted	Single-site, university-affiliated, military treatment facility in Florida	Non-comparative study evaluating the type and acceptance rate of student interventions (b)	The average amount of interventions during the eight-week rotation per student was 14.3; 95.6% of interventions were accepted
Leong et al. (2012) [18]	Hospital hemodialysis unit team consisting of physician, pharmacist, nurse, resident pharmacist, and student pharmacist. Twice-weekly nephrology teaching rounds and once-weekly interprofessional rounds	Non-US, single-site, teaching hospital	Qualitative assessment (observation) on the interactions of student pharmacists learning in a near-peer environment (a)	Four themes were noted: importance of organization and time management, recognition of a unique hierarchy, opportunity for cognitive congruence, and emergence of culture learning
Lyons et al. (2013) [19]	Inpatient colorectal surgery team consisting of physician, pharmacist, nurse, physician fellow, resident physician, student physician, student nurse, and student pharmacist. Eight or nine rounding sessions per semester	Single-site, university-affiliated hospital in Pennsylvania	Qualitative assessment (structured observation, debriefing interview) to assess the successful implementation and acceptance of interprofessional bedside rounding (a)	Observation indicated that students were engaged in rounds; the debriefing interviews indicated a high level of satisfaction with the experience
Maldonado et al. (2013) [20]	Solid organ transplant teams consisting of physicians, pharmacists, nurses, social workers, dieticians, financial coordinators, donor advocates, chaplains, and student pharmacists. Frequency or duration of rounds not noted	Multi-site, university-affiliated or teaching, tertiary-care hospitals	Pre-/post-survey assessing perceptions of interprofessional roles, communication, and teamwork (a)	Scores increased for 17 of the 22 survey items; positive changes were seen in the areas of roles and responsibilities, interprofessional communication, and teams and teamwork
Nwaesei et al. (2019) [21]	Inpatient internal medicine team consisting of physician, student physician, and student pharmacist. Daily rounds	Single-site, non-teaching, community hospital in Georgia	Pre-/post-experience surveys assessing student perceptions of physician and pharmacist collaboration (a)	Significant improvement in three interprofessional competency areas: value and ethics, roles and responsibilities, and teams and teamwork
Okoro et al. (2021) [22]	Multiple inpatient internal medicine specialty teams consisting of physician, student physician, and student pharmacist. Three-day-a-week rounds, for approximately four hours per day	Non-US, multi-site, university-affiliated hospitals	Pre-/post-qualitative focus group study (a)	Three themes were identified: level of preparedness and hospital environment, integration of knowledge and application to patient care, and interprofessional relationships and professional identity. They also noted unsatisfactory experiences and unmet expectations
Pastakia et al. (2011) [23]	Composition of inpatient interprofessional internal medicine team was not specified. Daily rounds	Non-US, single-site, teaching hospital	Retrospective cohort comparing clinical activities between American and Kenyan students (b)	Kenyan students completed more interventions than American students (16.7 vs 12.0 per day). They also had more consultations on HIV and antibiotics
Petrie (2011) [24]	Multiple inpatient surgical specialty teams consisting of physician, pharmacist, physician assistant, nurse practitioner, nurse, case manager, dietician, physical therapist, and student pharmacist. Daily rounds	Single-site, university-affiliated trauma center in Wyoming	Observational study of post-rotation ability-based outcomes, preceptor evaluation, and student self-evaluation (a)	Areas of needed improvement were assessed and included providing recommendations in a timely manner, self-confidence, identifying opportunities to communicate with team members, and addressing insecurities when answering drug information questions
Pham (2006) [25]	Inpatient internal medicine team consisting of physician, resident physician, student physician, student physician assistant, and student pharmacist. Daily rounds for a maximum of four hours per day	Single-site, community-teaching hospital in New York	Non-comparative study evaluating the type, quantity, and acceptance of student interventions (b)	Student pharmacists provided 28.8% of all interventions made by their pharmacy, with an acceptance rate of 92%. The most common intervention was providing drug information (46.8%)
Polo et al. (1994) [26]	Combination of acute-care geriatric unit and long-term care team consisting of physician, resident physician, resident pharmacist, nurse, dietician, social worker, student physician, and student pharmacist. Acute-care rounds four days per week and long-term care rounds twice weekly	Single-site, community-teaching hospital in Florida	Retrospective cohort comparing students' evaluation of the rotation, comparing students who completed a traditional long-term care rotation as compared to the integrated geriatric care rotation (a)	Students completing the integrated geriatric care rotation had consistently higher mean scores on rotation evaluation questions that assessed the value and variety of experience
Sweeney et al. (2000) [27]	Outpatient family medicine team consisting of resident physician and student pharmacist. Daily rounds that ranged from four to seven hours each day	Multi-site, university-affiliated, ambulatory care clinics in Ohio	Non-comparative study evaluating the type and quantity of student interventions (b)	Students averaged 16 interventions per week; the most common intervention was providing drug information
Vinluan et al. (2018) [28]	Inpatient internal medicine team consisting of physician, pharmacist, resident physicians, student physicians, and student pharmacists	Single-site, university-affiliated hospital in Texas	Non-comparative study evaluating the type and quantity of student interventions (b)	Students averaged 44 interventions over a six-week period; physician acceptance rate was 87%
Walker et al. (2010) [29]	Inpatient internal medicine team consisting of physician, pharmacist, resident physician, and student pharmacist. Daily rounds	Single-site, university-affiliated tertiary-care hospital in Michigan	Retrospective cohort evaluating the impact on site productivity before and after student incorporation; post-experience student and preceptor evaluations (a, b)	Students increased the number of patients assessed from 31-42% of eligible patients to 63-83% of eligible patients. The student experience survey had high mean scores, and students met or exceeded all performance expectations

All studies were published in peer-reviewed journals with a range of journal impact factors (0.5-5.6) [10-29]. The studies were published between 1994 and 2022, with the majority of studies (55%) published between 2010 and 2019 [10-29]. None of the studies specified a funding source. The type of hospital setting was described in 14 of the manuscripts. The majority (80%) of hospitals were described as a university (11/20) or teaching hospital (5/20) [10,13,16,18-19,24,26,28-29]. Only one hospital self-described as a non-academic, community hospital [21]. Only four studies specifically mentioned the type of community in which the hospital was located [15,21,26,29]. A review of locations based on author information found that all were located in urban or suburban area [10-29]. None were located in rural regions, although some described resource-limited urban locations [22-23].

All of the assessments performed were quasi-experimental. The most common evaluation methods were a non-comparative study design that described the type and amount of student interventions (eight), pre-/post-survey studies (four), and retrospective cohort studies (three) [10-13,15-17,20,24-29]. A review of the outcomes found that all of the studies' outcomes could be grouped into two overarching categories: the benefits of interprofessional rounding to student pharmacist learning or satisfaction and the benefits of student pharmacist participation on interprofessional rounds to patient care. Ten studies evaluated the benefits of interprofessional rounding on student learning or satisfaction (50%), nine studies evaluated the benefit of students on rounds to patient care (45%), and one study evaluated both (5%) [10-29]. The benefits of interprofessional rounding on student pharmacist learning or satisfaction were assessed by a variety of different methods, such as qualitative analysis, surveys, and student ability assessments [22,24,26]. In contrast, the benefit of student pharmacist participation in interprofessional rounds on patient care was assessed solely by a review of clinical intervention type and quantity [10-12,14,17,23,25,27]. Of these studies, only three utilized a comparison group [14,23,27].

Thirteen (68%) of the studies described the frequency of student pharmacist participation on interprofessional rounds. Of these studies, eight described daily rounding, and five described non-daily rounding [11,14,16,18-19,21-27]. The non-daily rounds ranged from less than weekly (8-9 sessions per semester) to four days per week [19,26]. There were four studies that quantified the hours that student pharmacists spent on interprofessional rounds, with a range of 3-7 hours [16,22,25,27]. Of the eight studies that described daily rounding, six (75%) assessed the impact of the student to patient care through the documentation of student interventions [14,23,25,27,29]. All of the five studies that described non-daily rounding assessed outcomes related to student learning, interprofessional competencies, or student acceptance of the activities [16,18-19,22,26]. Nineteen of the 20 studies described the interprofessional rounding team [10-22,24-29]. The most common participants, outside of the student pharmacists, were attending-level physicians (95%), resident physicians (63%), and pharmacists (58%) [10-22,24-29].

Discussion

This is the first review that focuses solely on the data supporting pharmacy students' participation on interprofessional rounds and provides a foundation for pharmacist preceptors who are interested in trying novel interprofessional rounding techniques. A recently published secondary analysis of a scoping review of inpatient interprofessional activities includes three of the studies included in our review [6]. However, the difference in search terms and date limits provides a distinction between the reviews, with this review a more focused assessment of student pharmacists on interprofessional rounding teams.

While there are reviews that describe the data for clinical rounding for medical students, there is a limited amount of published information regarding student pharmacist participation in interprofessional rounds [7]. In anticipation of a limited amount of data, an expansive definition of interprofessional rounding, defined as the pharmacy student and one other profession, and a scoping review strategy were utilized. Even with these options, our search found only 20 studies, with the majority of studies originating from university and teaching hospitals. The lack of data from smaller, community, and rural hospitals could be due to a difference in the type of interprofessional interactions done at those facilities or could reflect a lack of resources to support research and publication. Because of the lack of available data, we do not know to what extent rounding is available as a learning experience for student pharmacists in non-academic practice settings. Practice sites with limited pharmacy staff or small patient volumes (e.g., critical access hospitals) are not represented in the available data. 

The overall quality of assessments completed was low, with all of the data derived from quasi-experimental studies and several lacking a comparison group. This lack of rigor is not just a concern with pharmacy studies and has been noted in data supporting interprofessional collaboration and education in general [5,30]. A Cochrane review on the benefits of interprofessional collaboration found a very limited number of randomized studies, which limited the conclusions [5]. Within the larger arena of interprofessional collaboration and education, there is a need for data from a wider variety of practice types and locations, as well as higher-quality data.

Even though ACPE standards do not mention rounding as a requirement for APPEs, the majority of studies that mentioned rounding schedules described daily rounding. This is not surprising, since many interprofessional teams utilize a daily rounding schedule, based on an acute-care physician rounding model. However, we also found several studies that described a non-daily rounding schedule. All of these studies provided data on student-focused outcomes, such as confidence, interprofessional competency, or satisfaction with the experience. Given this information, there could be flexibility in rounding schedules, depending on the goals of the site and preceptor. Future research into the reasoning behind the preceptor's choice of a rounding schedule will help clarify why a certain schedule is utilized. In addition, increasing awareness of non-daily and non-traditional rounding options may increase the confidence of pharmacists at smaller or rural sites to provide learning opportunities to student pharmacists.

Another area of future study is the balance between the pros and cons of participating in interprofessional rounds. Is there a point at which the stress of preparing for and participating in rounds outweighs the benefit that the students gain by participating in rounds? How much do students hone their communication skills, teamwork skills, and application of clinical knowledge during activities outside of interprofessional rounds? Students may reach a maximum threshold of interventions performed; is there also a threshold at which the benefit of participating in rounds is outweighed by possible drawbacks, such as stress, burnout, and inability to participate in other clinical activities [11]? Continued research with a focus on student learning outcomes is needed.

## Conclusions

There are few studies that describe student pharmacist participation on interprofessional rounds. This review found that the outcomes studied can be divided into benefits to the student and benefits to the site or patient. There is a need for more high-quality studies to determine whether there is an optimal interprofessional rounding schedule. Future research should prioritize data from small, community hospitals.

## Appendices

Search string for MEDLINE database

MEDLINE search strategy performed: 5/9/2023 (separately by both authors)

("teaching rounds" [MeSH Terms] OR "round*, teaching" [tiab] OR "clinical round*" [tiab] OR "round*, clinical" [tiab] OR "morning report*" [tiab] OR "report*, morning" [tiab] OR "morning round*" [tiab] OR "round*, morning" [tiab] OR "attending round*" [tiab] OR "round*, attending" [tiab] OR "bedside round*" [tiab] OR  "bed-side round*" [tiab] OR "training round*" [tiab] OR "education* round*" [tiab] OR "round*, ward" [tiab] OR "ward round*" [tiab]) AND ("students, pharmacy" [MeSH Terms] OR "education, pharmacy" [MeSH Terms] OR "student pharmacist*" [tiab] OR "student*, pharmacy" [tiab] OR "student pharmacist*" [tiab])
